# The Epigenetic Regulation of Quiescent in Stem Cells

**DOI:** 10.1055/s-0043-1777072

**Published:** 2023-11-22

**Authors:** Mehran Radak, Hossein Fallahi

**Affiliations:** 1 Department of Biology, School of Sciences, Razi University, Baq-e-Abrisham, Kermanshah, Islamic Republic of Iran

**Keywords:** epigenetic regulation, quiescent stem cells, histone modifications, DNA methylation, molecular signature, environmental cues, tissue repair

## Abstract

This review article discusses the epigenetic regulation of quiescent stem cells. Quiescent stem cells are a rare population of stem cells that remain in a state of cell cycle arrest until activated to proliferate and differentiate. The molecular signature of quiescent stem cells is characterized by unique epigenetic modifications, including histone modifications and deoxyribonucleic acid (DNA) methylation. These modifications play critical roles in regulating stem cell behavior, including maintenance of quiescence, proliferation, and differentiation. The article specifically focuses on the role of histone modifications and DNA methylation in quiescent stem cells, and how these modifications can be dynamically regulated by environmental cues. The future perspectives of quiescent stem cell research are also discussed, including their potential for tissue repair and regeneration, their role in aging and age-related diseases, and their implications for cancer research. Overall, this review provides a comprehensive overview of the epigenetic regulation of quiescent stem cells and highlights the potential of this research for the development of new therapies in regenerative medicine, aging research, and cancer biology.

## Introducing Quiescent Stem Cells

Stem cells are cells that have the ability to self-renew and differentiate into different cell types. They are critical for the maintenance and repair of various tissues in the body. One unique characteristic of stem cells is their ability to enter a state of quiescence, or a dormant state, in which they stop actively dividing and remain in a state of inactivity until activated to differentiate. 1 2 3 Quiescence is a highly regulated and reversible state, and it plays an important role in the maintenance of stem cell pools in different tissues. Quiescent stem cells have been identified in many different tissues, including the hematopoietic system, the intestinal epithelium, and the skin. 4 5 The ability of stem cells to enter and exit quiescence is critical for their function. For example, in the hematopoietic system, quiescent hematopoietic stem cells (HSCs) are responsible for the long-term maintenance of blood cell production. When the demand for blood cells increases, quiescent HSCs can be activated to differentiate and produce more blood cells. 6 7 Quiescent stem cells are also thought to play a role in protecting stem cells from damage and preventing the accumulation of deoxyribonucleic acid (DNA) mutations. By entering a dormant state, stem cells can reduce the risk of acquiring DNA damage during cell division. 8 The regulation of stem cell quiescence is a complex process that involves many different factors, including signaling pathways, epigenetic modifications, and metabolic pathways. Understanding the mechanisms that regulate quiescence in stem cells is critical for the development of strategies to manipulate stem cell behavior for therapeutic purposes. 9

**Figure 1.**
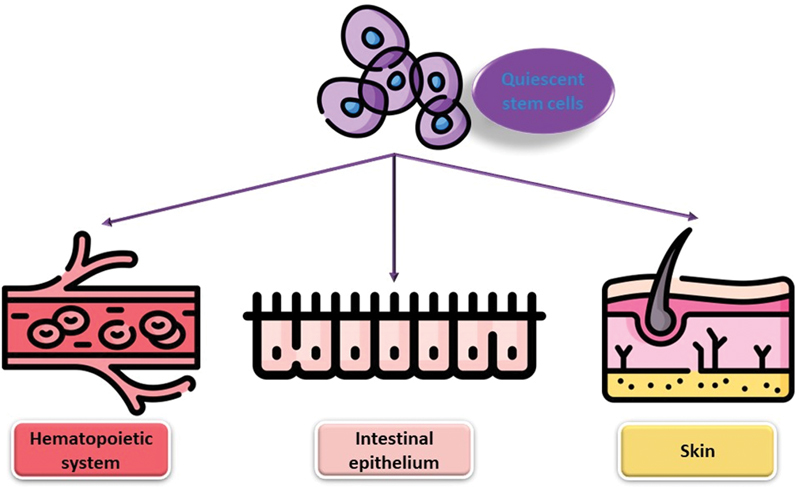
Presence of quiescent stem cells in many different tissues, including the hematopoietic system, the intestinal epithelium, and the skin.

Quiescence is an important state for stem cells, which allows them to maintain their stem cell properties and respond to changing physiological demands. Further research into the regulation of quiescence in stem cells will be essential for the development of new therapies for a range of diseases and conditions ( Fig. 1 ).

## Molecular Signature of Quiescent Stem Cells

Quiescent stem cells are a unique and important population of cells that play a critical role in tissue maintenance and regeneration. These cells have been found to have a distinct molecular signature that sets them apart from other stem cell populations. 10 11 One of the hallmarks of quiescent stem cells is their ability to enter a dormant state, in which they stop dividing and remain inactive until they are needed for tissue repair or regeneration. This state of quiescence is maintained by a complex network of signaling pathways, epigenetic modifications, and metabolic processes that work together to regulate stem cell behavior. 12 13 At the molecular level, quiescent stem cells have been found to exhibit unique gene expression patterns compared with other stem cell populations. For example, a study of quiescent HSCs found that they express high levels of genes involved in cell cycle arrest and DNA repair, while genes associated with cell proliferation and differentiation were downregulated. 11 14 In addition to gene expression changes, quiescent stem cells also exhibit epigenetic modifications that help to regulate their behavior. For example, quiescent HSCs have been found to have high levels of DNA methylation, which is thought to contribute to the maintenance of their stem cell properties. 15 16 Another important aspect of the molecular signature of quiescent stem cells is their metabolic profile. Quiescent stem cells have been found to have lower rates of metabolism compared with proliferating cells, which may help to protect them from oxidative stress and DNA damage. 17

**Figure 2.**
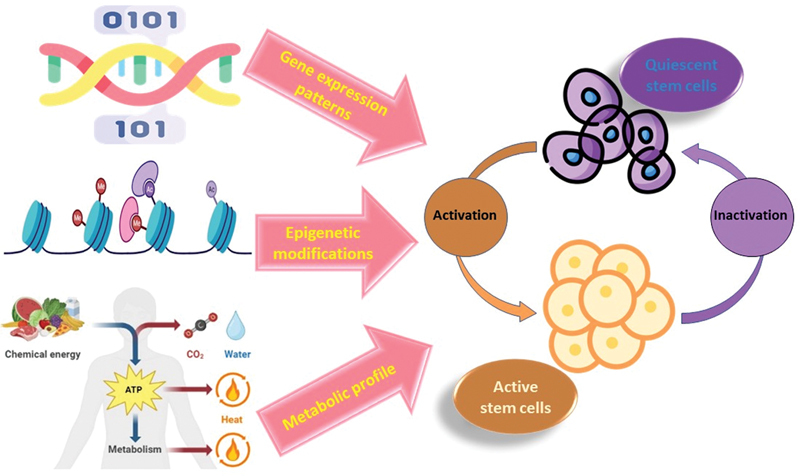
Molecular signature of quiescent stem cells. Quiescent stem cells have been found to exhibit unique gene expression patterns, epigenetics modifications and metabolic profile compared to other stem cell populations.

Overall, the molecular signature of quiescent stem cells is a complex and highly regulated network of gene expression, epigenetic modifications, and metabolic processes that work together to regulate stem cell behavior. Understanding the molecular mechanisms that govern quiescence in stem cells is critical for the development of new therapies for a range of diseases and conditions ( Fig. 2 ).

## Epigenetics Modifications in Quiescent Stem Cells

Epigenetic modifications play a critical role in regulating the behavior of quiescent stem cells. These modifications refer to changes in gene expression that occur without changes to the underlying DNA sequence, and they can be influenced by a variety of environmental factors. 18 One of the most well-studied epigenetic modifications in quiescent stem cells is DNA methylation. This modification involves the addition of a methyl group to cytosine residues in the DNA, which can lead to changes in gene expression. 19 In quiescent stem cells, DNA methylation has been found to be important for the maintenance of stem cell properties and the regulation of cell cycle progression. 20 For example, DNA methylation has been shown to regulate the expression of key cell cycle regulators such as cyclin D and p21, which are important for the transition from quiescence to proliferation. 21 Another important epigenetic modification in quiescent stem cells is histone modification. Histones are proteins that help to package DNA into chromatin, and modifications to these proteins can influence gene expression. 22 In quiescent stem cells, histone modifications have been found to play a critical role in the maintenance of stem cell properties and the regulation of differentiation. For example, histone acetylation has been shown to promote the expression of stem cell genes, while histone deacetylation can promote differentiation. 23 24 In addition to DNA methylation and histone modification, other epigenetic modifications such as chromatin remodeling and noncoding RNA regulation have also been found to be important in regulating quiescent stem cell behavior. These modifications can influence gene expression and help to maintain stem cell properties, as well as regulate the transition from quiescence to proliferation. 9 25

**Figure 3.**
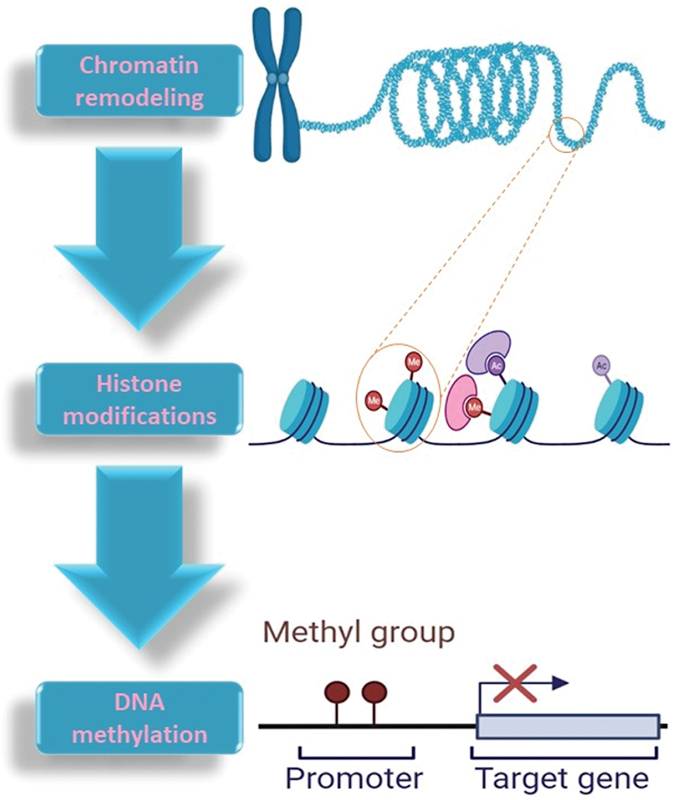
Epigenetic modifications play a critical role in regulating the behavior of quiescent stem cells.

Epigenetic modifications play a critical role in regulating the behavior of quiescent stem cells. Understanding the mechanisms that govern these modifications will be essential for the development of new therapies that target quiescent stem cells for tissue repair and regeneration ( Fig. 3 ).

## Histone Modifications

Histone modifications are critical epigenetic regulators of gene expression in quiescent stem cells. In quiescent cells, histone modifications help to maintain stem cell properties and regulate the transition from quiescence to proliferation. 26 27 Studies have shown that histone modifications such as acetylation, methylation, and phosphorylation play important roles in quiescent stem cells. For example, histone acetylation has been found to be important for maintaining the self-renewal capacity of HSCs. 28 In contrast, histone deacetylation has been shown to promote differentiation in neural stem cells. 29 Histone methylation has also been implicated in the regulation of quiescent stem cells. For example, the methylation of histone H3K4 has been shown to be important for maintaining the quiescent state of HSCs. 30 Similarly, the methylation of histone H3K27 has been found to play a critical role in regulating the transition from quiescence to proliferation in neural stem cells. 31 In addition to acetylation and methylation, histone phosphorylation has also been shown to play a role in quiescent stem cells. For example, the phosphorylation of histone H3 at serine 10 has been found to be important for the transition of quiescent HSCs to an active state. 32

Histone modifications are important regulators of gene expression in quiescent stem cells. Understanding the specific mechanisms by which these modifications regulate stem cell behavior will be critical for the development of new therapies for tissue repair and regeneration.

## Chromatin Remodeling

Chromatin remodeling is an important process in the regulation of gene expression in quiescent stem cells. Chromatin remodeling involves the physical reorganization of chromatin structure, which can influence the accessibility of DNA to transcription factors and other regulatory proteins. 33 In quiescent stem cells, chromatin remodeling plays a critical role in maintaining stem cell properties and regulating the transition from quiescence to proliferation. 34 For example, studies have shown that the adenosine triphosphate-dependent chromatin remodeling complex SWI/SNF is important for the maintenance of HSC quiescence. 35 SWI/SNF regulates the accessibility of DNA by altering chromatin structure, which in turn affects the binding of transcription factors to DNA. 36 Similarly, the polycomb group (PcG) proteins have been implicated in the regulation of chromatin structure in quiescent stem cells. PcG proteins are involved in the formation of repressive chromatin structures, which can regulate gene expression by inhibiting the accessibility of DNA to transcription factors. 37 Studies have shown that PcG proteins play an important role in the maintenance of quiescence in HSCs and neural stem cells. 38 In addition to SWI/SNF and PcG proteins, other chromatin remodeling complexes have also been implicated in the regulation of quiescent stem cells. For example, the nucleosome remodeling and deacetylase (NuRD) complex has been found to play a role in the maintenance of quiescence in HSCs. 39

Chromatin remodeling is an important mechanism for regulating gene expression in quiescent stem cells. Understanding the specific mechanisms by which chromatin remodeling complexes regulate stem cell behavior will be critical for the development of new therapies for tissue repair and regeneration.

## DNA Methylation

DNA methylation is a critical epigenetic modification that regulates gene expression in quiescent stem cells. DNA methylation involves the addition of a methyl group to the cytosine residues of DNA, usually in the context of a CpG dinucleotide. This modification can influence gene expression by altering the accessibility of DNA to transcription factors and other regulatory proteins. 40 41 In quiescent stem cells, DNA methylation plays an important role in regulating stem cell properties and the transition from quiescence to proliferation. 27 For example, studies have shown that DNA methylation is critical for the maintenance of HSC quiescence. Loss of DNA methylation leads to the activation of genes that promote proliferation, leading to a loss of stem cell self-renewal capacity. 20 42 To the maintenance of quiescence, DNA methylation also plays a role in regulating stem cell differentiation. Studies have shown that DNA methylation patterns are dynamically regulated during the differentiation of stem cells into different cell types. 43 Interestingly, recent studies have shown that DNA methylation patterns in quiescent stem cells are not static, but rather can be dynamically regulated by environmental cues. For example, changes in nutrient availability or exposure to toxins can alter DNA methylation patterns in HSCs, leading to changes in stem cell behavior. 44 45

Overall, DNA methylation is an important regulator of gene expression in quiescent stem cells. Understanding the specific mechanisms by which DNA methylation regulates stem cell behavior will be critical for the development of new therapies for tissue repair and regeneration.

## Future Perspective

The study of quiescent stem cells is a rapidly developing field with exciting future perspectives. One area of research that holds great promise is the development of new therapies for tissue repair and regeneration. Quiescent stem cells have the potential to differentiate into a variety of cell types, making them ideal candidates for cell-based therapies to treat a wide range of diseases and injuries. One challenge in the field of quiescent stem cells is the development of methods to activate quiescent stem cells and promote their proliferation and differentiation. A better understanding of the epigenetic modifications and signaling pathways that regulate quiescence and activation will be critical for developing such methods. Another area of research with future perspectives is the role of quiescent stem cells in aging and age-related diseases. Aging is associated with a decline in stem cell function and a decrease in the number of quiescent stem cells. Understanding the mechanisms that regulate stem cell quiescence and how these mechanisms are disrupted with age may lead to new therapies to improve stem cell function in aging populations. The study of quiescent stem cells also has implications for cancer research. Quiescent cancer stem cells have been implicated in tumor recurrence and resistance to chemotherapy. Understanding the mechanisms that regulate quiescence in cancer stem cells may lead to the development of new therapies to target these cells and improve cancer treatment outcomes.

**Figure 4.**
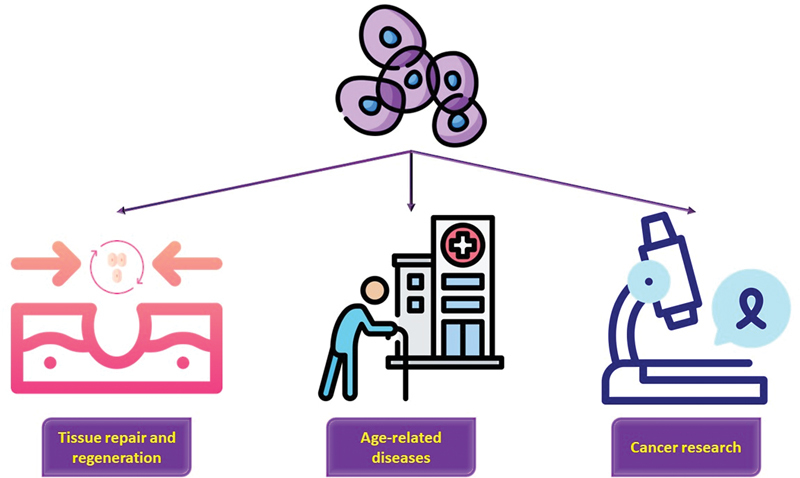
The study of quiescent stem cells has broad implications for regenerative medicine, aging research, and cancer biology.

The study of quiescent stem cells has broad implications for regenerative medicine, aging research, and cancer biology. Continued research in this field will undoubtedly lead to new insights and developments that have the potential to transform medicine and improve human health ( Fig. 4 ).

## References

[1] Chowdhury S Ghosh S Chowdhury S Ghosh S Stem cells an overview. Stem cells Biol Ther 2021 1 21

[2] Stripp B R Reynolds S D Maintenance and repair of the bronchiolar epithelium Proc Am Thorac Soc 2008 5 03 328 333 18403328 10.1513/pats.200711-167DR PMC2645243

[3] van Velthoven C TJ Rando T A Stem cell quiescence: dynamism, restraint, and cellular idling Cell Stem Cell 2019 24 02 213 225 30735649 10.1016/j.stem.2019.01.001 PMC6413865

[4] de Morree A Rando T A Regulation of adult stem cell quiescence and its functions in the maintenance of tissue integrity Nat Rev Mol Cell Biol 2023 24 05 334 354 36922629 10.1038/s41580-022-00568-6 PMC10725182

[5] Goodell M A Nguyen H Shroyer N Somatic stem cell heterogeneity: diversity in the blood, skin and intestinal stem cell compartments Nat Rev Mol Cell Biol 2015 16 05 299 309 25907613 10.1038/nrm3980 PMC5317203

[6] Marqués-Torrejón MÁ Williams C AC Southgate B LRIG1 is a gatekeeper to exit from quiescence in adult neural stem cells Nat Commun 2021 12 01 2594 33972529 10.1038/s41467-021-22813-w PMC8110534

[7] Arai F Suda T Maintenance of quiescent hematopoietic stem cells in the osteoblastic niche Ann N Y Acad Sci 2007 1106 01 41 53 17332071 10.1196/annals.1392.005

[8] Vitale I Manic G De Maria R Kroemer G Galluzzi L DNA damage in stem cells Mol Cell 2017 66 03 306 319 28475867 10.1016/j.molcel.2017.04.006

[9] Tümpel S Rudolph K L Quiescence: good and bad of stem cell aging Trends Cell Biol 2019 29 08 672 685 31248787 10.1016/j.tcb.2019.05.002

[10] Trentesaux C Striedinger K Pomerantz J H Klein O D From gut to glutes: the critical role of niche signals in the maintenance and renewal of adult stem cells Curr Opin Cell Biol 2020 63 88 101 32036295 10.1016/j.ceb.2020.01.004 PMC7247951

[11] Morizur L Chicheportiche A Gauthier L R Daynac M Boussin F D Mouthon M-A Distinct molecular signatures of quiescent and activated adult neural stem cells reveal specific interactions with their microenvironment Stem Cell Reports 2018 11 02 565 577 29983386 10.1016/j.stemcr.2018.06.005 PMC6092681

[12] Cheung T H Rando T A Molecular regulation of stem cell quiescence Nat Rev Mol Cell Biol 2013 14 06 329 340 23698583 10.1038/nrm3591 PMC3808888

[13] Lee S A Li K N Tumbar T Stem cell-intrinsic mechanisms regulating adult hair follicle homeostasis Exp Dermatol 2021 30 04 430 447 33278851 10.1111/exd.14251 PMC8016714

[14] Beerman I Seita J Inlay M A Weissman I L Rossi D J Quiescent hematopoietic stem cells accumulate DNA damage during aging that is repaired upon entry into cell cycle Cell Stem Cell 2014 15 01 37 50 24813857 10.1016/j.stem.2014.04.016 PMC4082747

[15] Ermolaeva M Neri F Ori A Rudolph K L Cellular and epigenetic drivers of stem cell ageing Nat Rev Mol Cell Biol 2018 19 09 594 610 29858605 10.1038/s41580-018-0020-3

[16] Nakhaei-Rad S Nakhaeizadeh H Götze S The role of embryonic stem cell-expressed RAS (ERAS) in the maintenance of quiescent hepatic stellate cells J Biol Chem 2016 291 16 8399 8413 26884329 10.1074/jbc.M115.700088 PMC4861415

[17] Mandal P K Blanpain C Rossi D J DNA damage response in adult stem cells: pathways and consequences Nat Rev Mol Cell Biol 2011 12 03 198 202 21304553 10.1038/nrm3060

[18] Liu L Cheung T H Charville G W Chromatin modifications as determinants of muscle stem cell quiescence and chronological aging Cell Rep 2013 4 01 189 204 23810552 10.1016/j.celrep.2013.05.043 PMC4103025

[19] Moore L D Le T Fan G DNA methylation and its basic function Neuropsychopharmacology 2013 38 01 23 38 22781841 10.1038/npp.2012.112 PMC3521964

[20] Chen Z Guo Q Song G Hou Y Molecular regulation of hematopoietic stem cell quiescence Cell Mol Life Sci 2022 79 04 218 35357574 10.1007/s00018-022-04200-w PMC11072845

[21] Karimian A Ahmadi Y Yousefi B Multiple functions of p21 in cell cycle, apoptosis and transcriptional regulation after DNA damage DNA Repair (Amst) 2016 42 63 71 27156098 10.1016/j.dnarep.2016.04.008

[22] Pepenella S Murphy K J Hayes J J Intra- and inter-nucleosome interactions of the core histone tail domains in higher-order chromatin structure Chromosoma 2014 123(1-2):3 13 23996014 10.1007/s00412-013-0435-8 PMC3938996

[23] Martynoga B Mateo J L Zhou B Epigenomic enhancer annotation reveals a key role for NFIX in neural stem cell quiescence Genes Dev 2013 27 16 1769 1786 23964093 10.1101/gad.216804.113 PMC3759694

[24] Yang J Tang Y Liu H Guo F Ni J Le W Suppression of histone deacetylation promotes the differentiation of human pluripotent stem cells towards neural progenitor cells BMC Biol 2014 12 95 25406762 10.1186/s12915-014-0095-z PMC4254204

[25] Wu H Sun Y E Epigenetic regulation of stem cell differentiation Pediatr Res 2006 59(4 Pt 2):21R 25R 16549544 10.1203/01.pdr.0000203565.76028.2a

[26] Frye M Fisher A G Watt F M Epidermal stem cells are defined by global histone modifications that are altered by Myc-induced differentiation PLoS One 2007 2 08 e763 17712411 10.1371/journal.pone.0000763 PMC1945016

[27] Luo M Li J-F Yang Q Stem cell quiescence and its clinical relevance World J Stem Cells 2020 12 11 1307 1326 33312400 10.4252/wjsc.v12.i11.1307 PMC7705463

[28] Yang Y Kueh A J Grant Z L The histone lysine acetyltransferase HBO1 (KAT7) regulates hematopoietic stem cell quiescence and self-renewal Blood 2022 139 06 845 858 34724565 10.1182/blood.2021013954

[29] Hsieh J Nakashima K Kuwabara T Mejia E Gage F H Histone deacetylase inhibition-mediated neuronal differentiation of multipotent adult neural progenitor cells Proc Natl Acad Sci U S A 2004 101 47 16659 16664 15537713 10.1073/pnas.0407643101 PMC527137

[30] Yang Z Shah K Khodadadi-Jamayran A Jiang H Control of hematopoietic stem and progenitor cell function through epigenetic regulation of energy metabolism and genome integrity Stem Cell Reports 2019 13 01 61 75 31231026 10.1016/j.stemcr.2019.05.023 PMC6627005

[31] Murao N Noguchi H Nakashima K Epigenetic regulation of neural stem cell property from embryo to adult Neuroepigenetics 2016 5 1 10

[32] Fagnocchi L Mazzoleni S Zippo A Integration of signaling pathways with the epigenetic machinery in the maintenance of stem cells Stem Cells Int 2016 2016 8.652748E6 10.1155/2016/8652748 PMC4699037 26798364

[33] Klemm S L Shipony Z Greenleaf W J Chromatin accessibility and the regulatory epigenome Nat Rev Genet 2019 20 04 207 220 30675018 10.1038/s41576-018-0089-8

[34] Sun S Jiang N Jiang Y Chromatin remodeler Znhit1 preserves hematopoietic stem cell quiescence by determining the accessibility of distal enhancers Leukemia 2020 34 12 3348 3358 32694618 10.1038/s41375-020-0988-5 PMC7685981

[35] Tu Z Zheng Y Role of ATP-dependent chromatin remodelers in hematopoietic stem and progenitor cell maintenance Curr Opin Hematol 2022 29 04 174 180 35787545 10.1097/MOH.0000000000000710 PMC9257093

[36] Krasteva V Crabtree G R Lessard J A The BAF45a/PHF10 subunit of SWI/SNF-like chromatin remodeling complexes is essential for hematopoietic stem cell maintenance Exp Hematol 2017 48 58 7.1E16 27931852 10.1016/j.exphem.2016.11.008 PMC11975438

[37] Atchison L Ghias A Wilkinson F Bonini N Atchison M L Transcription factor YY1 functions as a PcG protein in vivo EMBO J 2003 22 06 1347 1358 12628927 10.1093/emboj/cdg124 PMC151054

[38] Lu Z Hong C C Kong G Polycomb group protein YY1 is an essential regulator of hematopoietic stem cell quiescence Cell Rep 2018 22 06 1545 1559 29425509 10.1016/j.celrep.2018.01.026 PMC6140794

[39] Bode D Yu L Tate P Pardo M Choudhary J Characterization of two distinct nucleosome remodeling and deacetylase (NuRD) complex assemblies in embryonic stem cells Mol Cell Proteomics 2016 15 03 878 891 26714524 10.1074/mcp.M115.053207 PMC4813707

[40] Angeloni A Bogdanovic O Enhancer DNA methylation: implications for gene regulation Essays Biochem 2019 63 06 707 715 31551326 10.1042/EBC20190030

[41] Miller J L Grant P A The role of DNA methylation and histone modifications in transcriptional regulation in humans Subcell Biochem 2013 61 289 317 23150256 10.1007/978-94-007-4525-4_13 PMC6611551

[42] Momparler R L Côté S Momparler L F Epigenetic modulation of self-renewal capacity of leukemic stem cells and implications for chemotherapy Epigenomes 2020 4 01 3 34968237 10.3390/epigenomes4010003 PMC8594708

[43] Cheng Y Xie N Jin P Wang T DNA methylation and hydroxymethylation in stem cells Cell Biochem Funct 2015 33 04 161 173 25776144 10.1002/cbf.3101 PMC4687961

[44] Ancel S Stuelsatz P Feige J N Muscle stem cell quiescence: controlling stemness by staying asleep Trends Cell Biol 2021 31 07 556 568 33674167 10.1016/j.tcb.2021.02.006

[45] Breton-Larrivée M Elder E McGraw S DNA methylation, environmental exposures and early embryo development Anim Reprod 2019 16 03 465 474 32435290 10.21451/1984-3143-AR2019-0062 PMC7234019

